# Has cross-level clinical coordination changed in the context of the pandemic? The case of the Catalan health system

**DOI:** 10.1186/s12913-024-11445-7

**Published:** 2024-08-21

**Authors:** Daniela Campaz-Landazabal, Ingrid Vargas, Elvira Sánchez, Francesc Cots, Pere Plaja, Joan Manuel Perez-Castejón, Antonio Sánchez-Hidalgo, María Luisa Vázquez

**Affiliations:** 1 Health Policy and Health Services Research Group, Health Policy Research Unit, Consortium for Health Care and Social Services of Catalonia, Barcelona, 08022 Spain; 2 Serveis de Salut Integrats Baix Empordà - Hospital de Palamós. Unitat d’Inf. Assistencial, Palamós, 17230 Spain; 3 Parc de Salut Mar, Barcelona, 08003 Spain; 4 Fundació Salut Empordà, Figueres, 17600 Spain; 5 Badalona Serveis Assistencials, Badalona, 08911 Spain; 6 Consorci Sanitari de Terrassa, Terrassa, 08227 Spain

**Keywords:** Clinical coordination, Care coordination, Coordination mechanisms, ICT-based coordination mechanism, Primary care, Secondary care, COVID-19, Pandemic

## Abstract

**Background:**

The COVID-19 pandemic triggered numerous changes in health services organisation, whose effects on clinical coordination are unknown. The aim is to analyse changes in the experience and perception of cross-level clinical coordination and related factors of primary (PC) and secondary care (SC) doctors in the Catalan health system between 2017 and 2022.

**Methods:**

Comparison of two cross-sectional studies based on online surveys by means of the self-administration of the COORDENA-CAT (2017) and COORDENA-TICs (2022) questionnaires to PC and SC doctors. Final sample *n* = 3308 in 2017 and *n* = 2277 in 2022. Outcome variables: experience of cross-level information and clinical management coordination and perception of cross-level clinical coordination in the healthcare area and related factors. Stratification variables: level of care and year. Adjusting variables: sex, years of experience, type of specialty, type of hospital, type of management of PC/SC. Descriptive bivariate and multivariate analysis using Poisson regressions models to detect changes between years in total and by levels of care.

**Results:**

Compared with 2017, while cross-level clinical information coordination remained relatively high, with a slight improvement, doctors of both care levels reported a worse experience of cross-level clinical management coordination, particularly of care consistency (repetition of test) and accessibility to PC and, of general perception, which was worse in SC doctors. There was also a worsening in organisational (institutional support, set objectives, time available for coordination), attitudinal (job satisfaction) and interactional factors (knowledge between doctors). The use of ICT-based coordination mechanisms such as shared electronic medical records and electronic consultations between PC and SC increased, while the participation in virtual joint clinical conferences was limited.

**Conclusions:**

Results show a slight improvement in clinical information but also less expected setbacks in some dimensions of clinical management coordination and in the perception of clinical coordination, suggesting that the increased use of some ICT-based coordination mechanisms did not counteract the effect of the worsened organisational, interactional, and attitudinal factors during the pandemic. Strategies are needed to facilitate direct communication, to improve conditions for the effective use of mechanisms and policies to protect healthcare professionals and services in order to better cope with new crises.

**Supplementary Information:**

The online version contains supplementary material available at 10.1186/s12913-024-11445-7.

## Introduction

Cross-level clinical coordination is a priority for health systems, particularly for national health systems (NHS) based on primary care (PC), such as the Catalan health system, as it contributes to care quality and efficiency [1, 2]. Among others, it is highly relevant for the care of patients with chronic conditions or complex needs, who frequently transit across different levels of care over time [3, 4]. It has been addressed in the last decades by a range of policies and organisational strategies to promote care coordination [5–7], which underwent a rapid transformation during the pandemic [8, 9].

Clinical coordination is here defined [10] as the harmonious connection of the different health services needed to provide care to a patient throughout the care continuum to achieve a common objective without conflicts and it is analysed based on a comprehensive theoretical framework [11, 12]. Two types are distinguished [12]: (a) clinical information coordination, that refers to the transfer and use of the patient clinical information between providers and (b) clinical management coordination, which involves healthcare provision in a sequential and complementary way and encompasses three dimensions: consistency of care, adequate patient follow-up and accessibility between levels. Clinical coordination is influenced, among others, by organisational factors, such as institutional support, the existing cross-level coordination mechanisms, time available to coordinate and integration of providers’ management [13] and interactional factors, such as knowledge between doctors or attitudinal factors, job satisfaction [13].

During the COVID-19 pandemic, several organisational changes were introduced in the health services, to reduce the risk of contagion while maintaining an appropriate response to the population healthcare needs [8, 9, 14], the effects of which on clinical coordination and its related factors is unknown. On the one hand, there was an acceleration in the introduction of information and communication technologies (ICT) based-coordination mechanisms [8, 15, 16], such as shared electronic medical records (EMR) or electronic consultations between PC and secondary care (SC) [8, 16], which should have increased cross-level clinical information coordination by facilitating transfer of information between levels [2] and clinical management coordination by improving access to SC and adequacy of referrals [17]. On the other hand, the cancellation of non-urgent treatments, tests, or consultations [8], especially during the first waves of the pandemic, and the introduction of telephone triage [8] and the rapid adoption of telemedicine [9, 18] opened new avenues of access to services that increased professionals workload, especially in PC [19], decreasing the time available to coordinate with the other level [8].

Despite its relevance, international evidence on cross-level clinical coordination during the pandemic is scarce. No methodological solid studies have been identified and the few reports mostly based on clinical records review of specific health services focused on evaluating one dimension of clinical coordination, accessibility between levels, reporting a global backlog in access especially to SC [20, 21]. The analysis of changes in factors influencing coordination is even more limited, and mostly explored by surveys to SC doctors that analysed job satisfaction during the pandemic [22, 23].

The Catalan NHS is part of the Spanish NHS, which is funded by taxes, of universal access and decentralised to the regions [24]. The provision of healthcare is organised into two care levels: PC, which acts as the gatekeeper and coordinator of the patient care throughout the care continuum, and SC that acts as a consultant to PC and is responsible for the management of more complex procedures [24]. in the Catalan NHS, patient care is the responsibility of a variety of providers: a large public entity, the Catalan Health Institute, and several public consortia, municipal foundations, and some private foundations (mostly non-profit but also some for-profit), which make up the Integrated Healthcare System for Public Use [25]. This diversity has originated differences in the type of management of PC and SC providers across the different healthcare areas (1) integrated: PC and SC providers are mostly managed by the same entity; (2) partially integrated: an entity manages SC and some PC centres, while the rest are managed by other entities, and (3) non-integrated: where PC and SC are managed by different entities [26, 27]. This complexity makes cross-level coordination even more relevant. Before the pandemic, the Catalan NHS had implemented several strategies, such as promoting integrated management or the introduction of a variety of coordination mechanisms, such as case managers, liaison nurses or shared protocols between levels, including ICT-based mechanisms, such as shared EMRs and electronic consultations between professionals [28], with great differences in implementation between areas and services [29]. Analysis on the changes during the pandemic in Spain or Catalonia are limited to few studies on accessibility of specific SC services that reported an increase in waiting times [30, 31] and a survey to PC doctors showing a low perception of clinical coordination between levels [32]. Regarding influencing factors, a study showed an increase in the use of electronic consultations [31] and a low satisfaction with the job was reported by a survey to SC doctors in training [33].

This study allowed us to explore whether there were any changes in cross-level clinical coordination and influencing factors in the Catalan health system during the pandemic and identify elements to guide strategies for improving it.

## Methods

### Aim

The aim of this study, which forms part of a wider research [34], is to analyse the changes in the experience, perception of cross-level clinical coordination of primary care (PC) and secondary care (SC) doctors and related factors in the Catalan health system between 2017 and 2022.

### Study design and areas of study

A comparative analysis was conducted of two cross-sectional studies based on the online surveys by means of the self-administration of the COORDENA-CAT (2017) and COORDENA-TICs (2022) questionnaires by primary (PC) and secondary care (SC) doctors of the Catalan National Health System. In both years, the areas of study were defined based on the primary healthcare areas (PCC) and their referral hospitals (acute, and in 2017 also long-term care).

### Study population and sample

The study population consisted of PC and SC doctors that had been working for at least one year in the organisation, and whose daily practice involved direct contact with patients, and with doctors of the other care level. Specialists in pathology, immunology, neurophysiology, radiology, pharmacy and clinical analysis and preventive medicine were excluded. In both surveys, the selection of the sample took place in two stages. In the first, 41 healthcare areas and its organisations belonging to the Catalan public health system were invited to participate (in 2022, starting by those that had already participated in 2017). In the second, those organisations that agreed to participate, sent an invitation with the questionnaire to all doctors who met the inclusion criteria. The final sample in 2017 was 3,308 doctors of 15,813 invited to participated (21% participation rate) and in 2022, a total of 2,277 doctors of 12,987 invited (17.5% participation rate). Of the 41 healthcare areas invited, in 2017, 32 areas participated both with primary care centres and hospitals, while in 2022, 22 areas did so.

### Questionnaire

In 2017, the COORDENA questionnaire, which had been developed following the theoretical framework underlying this study [35] was adapted, pre-tested, piloted, and validated for the Catalan context [36]. It consists of three main sections: the first measures doctors’ experience of cross-level clinical information and clinical management coordination and perception of coordination within the healthcare area, by means of 12 items (described in detail in the variables of analysis section) and using a Likert scale and two open-ended questions on their reasons for that perception and suggestions for improvement. The second section measures the availability and use of cross-level clinical coordination mechanisms (shared electronic medical records (EMR), electronic consultations, telephone consultations, email consultations, joint clinical case conference, liaison nurses, shared protocols, and guidelines); and the third, individual, organisational and interactional factors that influence clinical coordination. In 2022, the questionnaire was slightly modified and renamed as COORDENA-TICs [34]. This new version keeps all main sections and all items of doctors’ experience of cross-level clinical information and clinical management coordination, perception of coordination and of factors related to clinical coordination. The adaptation relies in the coordination mechanisms section, that focuses on ICT-based coordination mechanisms but maintaining the items. Additional items related to the pandemic and some characteristics of use of some mechanisms were included but were not analysed in this study [Additional file 1].

### Data collection

Data collection for the first survey took place between October and December in 2017 and for the second, between May and June of 2022 and October 2022 to April 2023. In both surveys, all PC and SC doctors who met the inclusion criteria of the organisations that agreed to participate, were invited to answer the questionnaire. To promote doctors’ participation, the involved organisations conducted informative sessions and displayed posters and posts in their institutional intranets. The invitation was sent to their corporate e-mail address, and included a link randomly generated that provided anonymous access to the questionnaire. The participants had the possibility of closing the incomplete questionnaire and retaking it on another occasion, and as many times as they wished, as long as it had not been sent.

### Variables of analysis

The outcome variables were (a) experience of cross-level coordination of clinical information (information transfer and use; 3 items) and of clinical management (consistency of care, adequate follow-up, and accessibility; 11 items); (b) general perception of cross-level coordination in the area (1 item); (c) organisational, interactional, and attitudinal related factors (8 items); (d) use of ICT-based coordination mechanisms (shared EMR of the region (HC3/HES) and of the organisation and, electronic consultations through EMR, telephone consultations, email consultations and joint clinical session through videoconference (in 2022) (12 items). The explanatory variables were *stratification variables*: year and level of care and, *adjustment variables*: (a) sociodemographic: sex, type of speciality (clinical and surgical/clinical-surgical), (b) employment characteristics: years working as a doctor (c) type of hospital (local/regional, high-resolution, high-technology); and type of area according to the management of PC/SC (integrated, partially integrated, non-integrated).

### Analysis

A bivariate descriptive analysis stratified by level of care (primary and secondary care) and year was conducted to determine the distribution of the outcome and explanatory variables. To identify differences between years in total and within the subgroups, the Chi-square test was used. To analyse the changes in the degree of clinical coordination and related factors including use of ICT-based coordination mechanisms between the two years in the total sample and within the levels of care, Poisson regression models with robust variance were estimated, obtaining prevalence ratios (PR) and their 95% confidence intervals (CI 95%), adjusting for the explanatory variables: level of care, sex, years working as a doctor, type of specialty, and type of hospital, in the case of the aggregated analysis of the sample. And sex, years working as a doctor, type of specialty, and type of hospital for the subgroup analysis. Type of area according to the management of PC/SC was used to control for a possible cluster effect. Participants who answered don’t know/no answer were excluded. Bayesian and Akaike reporting criteria were used to assess the fitness of the models.

Missing values were low for the outcome variables related to the experience and perception of clinical coordination (0.66–7.26%) and those related to factors and use of coordination mechanisms varied from 2.23 to 17.35% and were at random [see Additional file 2]. Percentage of missing values for explanatory variables varied from 0 to 19.46% and were at random [see Additional file 2]. A full case analysis to manage missing values was adopted. To make the results more robust, sensitivity analyses were performed to evaluate two alternative scenarios (1) analysis of the data after doing multiple imputation of those variables with a percentage of missing values higher than 10% [see Additional file 2] and (2) analysis of the data including the participants who answered do not know/no answer to the questions [see Additional file 3]. In both cases, there were not significant differences with the results presented in this article. Finally, a content analysis was performed for the open-ended questions on reasons for the general perception of cross-level clinical coordination in the healthcare area and suggestions for improvement in 2022. The answers were coded and classified into categories. Frequencies were calculated and presented stratified by level of care. Statistical analyses were conducted using Stata v.15.

## Results

### Characteristics of the sample

The sample composition was similar in both study years, with some differences. In 2022, most doctors still were women, but the proportion slightly decreased in PC (68.51% in 2017 to 66.50%) and increased in SC (51.76–53.62%) and in both levels most doctors still were between 41 and 55 years of age. Most doctors in both levels had clinical specialities, but the proportion of doctors with surgical (9.23% in 2017 to 14.85%) and medical/surgical (12.37% in 2017 to 23.31%) specialities considerably increased in SC and dropped almost to zero in PC [Table 1]. Regarding employment characteristics, one third of SC doctors still had 11–20 years’ work experience and 6 to 15 years working in the organisation (30.09% and 31.34% respectively), while in the case of PC, there was an increase of the doctors who had 21 to 30 years’ work experience (30.67% in 2017 to 38.79%) and 16 to 25 years working in the organisation (33.58% in 2017 to 37.22%). The proportion of doctors with a permanent contract increased in SC (88.10% in 2017 to 92.96%) and decreased in PC (96.31% in 2017 to 93.57%) and in both levels increased those with a full-time contract (PC: 92.71% in 2017 to 96.40%; SC: 91.44% in 2017 to 94.25%). Finally, regarding the type of area, the highest proportion of doctors worked in an area where the same entity manages SC and the majority of PC (44,04% in 2017 and 46,60% in 2022) and this proportion increased in SC (45.22% in 2017 to 50.45%). In terms of the type of hospital, the proportion of doctors working on an area with a high-technology hospital almost doubled (19.38 in 2017 to 38.21%) [Table 1].

**Table 1:** Description of the sample years 2017 and 2022. Total and, by level of care

	Total		Primary Care		Secondary Care	
	2017 *N* = 3308	2022 *N* = 2277	2017 *N* = 1141	2022 *N* = 945	2017 *N* = 2167	2022 *N* = 1332
	*n* (%)	*n* (%)	*n* (%)	*n* (%)	*n* (%)	*n* (%)
***Sociodemographic characteristics***						
Sex						
Man	1.214(41.86)	740(39.57) *	307(30.79)	251(32.22)	907(47.66)	489(44.82) *
Woman	1.668(57.52)	1.103(58.98) *	683(68.51)	518 (66.50)	985(51.76)	585(53.62) *
Age						
25–40 years	776(28.20)	453(25.03) *	208(21.92)	124(16.45) *	568 (31.50)	329(31.16) *
41–55 years	1.279(46.48)	859(47.46) *	488(51.42)	405(53.71) *	791(43.87)	454(42.99) *
56–70 years	697(25.33)	498(27.51) *	253(26.66)	225(29.84) *	444(24.63)	273(25.85) *
Country of birth						
Spain	2.469(85.58)	1.544(83.06) *	858(86.49)	638(82.32) *	1.611(85.10)	906(83.58) *
Other	357(12.37)	250(13.45) *	115(11.59)	113(14.58) *	242 (12.78)	137(12.64) *
Medical speciality						
Clinical speciality	2.189(79.51)	1.458(77.43)	788(81.57)	771(99.87) *	1.401(78.40)	687 (61.84) *
Surgical speciality	225(8.17)	165(8.76)	60(6.21)	0(0) *	165(9.23)	165 (14.85) *
Medical and surgical speciality	339(12.31)	260(13.81)	118(12.22)	1(0.13) *	221(12.37)	259 (23.31) *
***Employment characteristics***						
Years working as a doctor						
0 to 10 years	480(17.20)	272(14.84) *	121(12.54)	77(10.09) *	359(19.67)	195(18.22)
11 to 20 years	921(33.01)	520(28.37) *	339(35.13)	198(25.95) *	582(31.89)	322(30.09)
21 to 30 years	790(28.32)	605(33.01) *	296(30.67)	296(38.79) *	494(27.07)	309(28.88)
31 to 50 years	599(21.47)	436(23.79) *	209(21.66)	192(25.16) *	390(21.37)	244(22.80)
Years working in the organisation						
1 to 5 years	438(15.97)	313(17.28) *	89(9.34)	96(12.72) *	349(19.50)	217(20.55) *
6 to 15 years	1.019(37.15)	497(27.44) *	324(34.00)	166(21.99) *	695(38.83)	331(31.34) *
16 to 25 years	733(26.72)	586(32.36) *	320(33.58)	281(37.22) *	413(23.07)	305(28.88) *
26 to 50 years	553(20.16)	415(22.92) *	220(23.08)	212(28.08) *	333(18.60)	203(19.22) *
Type of contract a)						
Permanent	2.630(90.94)	1.744(93.21) *	965(96.31)	728(93.57) *	1.665(88.10)	1.016(92.96) *
Temporary	262(9.06)	127(6.79) *	37(3.69)	50(6.43) *	225(11.90)	77(7.04) *
Type of contract b)						
Full-time	2.660(91.88)	1.781(95.14) *	929(92.71)	749(96.40) *	1.731(91.44)	1.032(94.25) *
Part-time	235(8.12)	91(4.86) *	73(7.29)	28(3.60) *	162(8.56)	63(5.75) *
***Type of area***						
Area according to the managing of PC/SC						
One entity manages SC and majority of PC	1.457(44.04)	1.061(46.60) *	477(41.81)	389(41.16)	980(45.22)	672(50.45) *
One entity manages SC and minority of PC	892(26.99)	546(23.98) *	354(31.03)	267(28.25)	538(24.83)	279(20.95) *
Different entities manage SC and PC	959(28.99)	670(29.42) *	310(27.17)	289(30.58)	649(29.95)	381(28.60) *
Area according to the type of hospital						
Local and regional hospitals	1.857(56.14)	911(40.01) *	770(67.48)	498(52.70) *	1.087(50.16)	413(31.01) *
High resolution regional hospitals	810(24.49)	496(21.78) *	222(19.46)	197(20.85) *	588(27.13)	299(22.45) *
High technology general hospitals	641(19.38)	870(38.21) *	149(13.06)	250(26.46) *	492(22.70)	620(46.55) *

PC: Primary Care, SC: Secondary care. * *p*-value < 0.05 for differences between years

### Changes in doctor’s experience and perception of cross-level clinical coordination

Compared with 2017, the degree of cross-level coordination of clinical information (transfer and use) experienced by doctors was still high in 2022, with a slight increase of those reporting that the information they share is needed for the patient clinical management (PR:1.07, CI 95% 1.03–1.10), which was higher among PC doctors (PR:1.08, CI 95% 1.03–1.12) [Table 2].

**Table 2:** Changes in the experience of cross-level clinical information and clinical management coordination and perception of clinical coordination between 2017 and 2022, in total and, by level of care

		Total			Primary care			Secondary care		
		2017 *N* = 3308	2022 *N* = 2277	2022/2017^a^ changes	2017 *N* = 1141	2022 *N* = 945	2022/2017^a^ changes	2017 *N* = 2167	2022 *N* = 1332	2022/2017^a^ changes
		n (%)^*b*^	n (%)^*b*^	a PR (CI 95%) ^*c*^	n (%)^*b*^	n (%)^*b*^	a PR (CI 95%) ^*d*^	n (%)^*b*^	n (%)^*b*^	a PR (CI 95%) ^*d*^
***Coordination of clinical information between levels of care***										
PC and SC doctors share information on the care of patients we have in common		1.959(63.11)	1.520(67.59)	1.06(0.94–1.20)	757(70.09)	694(74.38)	1.09(0.93–1.28)	1.202(59.39)	826(62.77)	1.06 (0.93–1.20)
The information we share is as required for the care of these patients		2.224(72.14)	1.755(78.49)	**1.07(1.03–1.10)**	830(76.92)	765(81.82)	**1.08 (1.03–1.12)**	1.394(69.56)	990(76.10)	**1.06(1.01–1.11)**
PC and SC doctors use the information that we share		2.489(81.77)	1.822(82.15)	0.99(0.97–1.01)	911(84.90)	793(85.82)	0.99(0.96–1.03)	1.578(80.06)	1.029(79.52)	0.99(0.97–1.02)
***Coordination of clinical management: Care consistency between levels of care***										
We agree with the treatments prescribed or directions given to the patients by doctors of the other level		2.357(77.99)	1.757(80.97)	**1.02(1.01–1.04)**	792(74.16)	743(80.06)	**1.09(1.06–1.12)**	1.565(80.09)	1.014(81.64)	0.99(0.96–1.01)
There are contraindications and/or duplications in the treatments prescribed by primary and secondary care doctors		920(30.46)	701(32.29)	1.03(0.99–1.07)	416(38.95)	370(39.83)	0.98(0.89–1.08)	504(25.82)	331(26.65)	**1.10(1.03–1.17)**
PC and SC doctors establish a treatment plan together for patients that require this		422(13.82)	269(12.37)	**0.90(0.83–0.97)**	129(11.99)	101(10.97)	0.93(0.86-1.00)	293(14.82)	168(13.40)	0.90(0.78–1.04)
We repeat the tests that doctors have already carried out at the other level		872(28.55)	733(33.27)	**1.19(1.06–1.32)**	299(28.02)	335(36.41)	**1.37(1.11–1.69)**	573(28.84)	398(31.02)	**1.09(1.07–1.12)**
***Coordination of clinical management: Adequate follow up between levels of care***										
PC doctors refer the patients to secondary care when appropriate		2.493(83.13)	1.786(84.29)	**0.98(0.96–0.99)**	1.060(99.07)	901(99.45)	0.99(0.99-1.00)	1.433(74.29)	885(72.96)	**0.96(0.92–0.99)**
SC doctors send the patients back to primary care for follow-up when appropriate		2.484(83.55)	1.700(80.88)	0.98(0.96-1.00)	811(76.65)	655(72.54)	**0.94(0.91–0.97)**	1.673(87.36)	1.045(87.16)	0.99(0.95–1.04)
SC doctors make recommendations to the primary care doctor on the follow-up of patients (diagnosis. treatment. other guidelines)		1.780(59.10)	1.287(60.51)	**1.06(1.03–1.08)**	421(39.38)	443(48.79)	**1.16(1.09–1.24)**	1.359(69.94)	844(69.24)	1.01(0.99–1.04)
PC doctors clarify any doubts on the follow-up of patients with the secondary care doctors		1.249(42.63)	984(47.13)	**1.10(1.05–1.15)**	588(55.00)	572(63.13)	**1.20(1.10–1.32)**	661(35.52)	412(34.86)	**1.05(1.04–1.07)**
***Coordination of clinical management: Accessibility between levels of care***										
On being referred in the normal way to secondary care. the patient does wait a long time to be seen		2.240(78.57)	1.639(79.99)	1.00(0.98–1.01)	1.057(98.42)	890(97.59)	0.99(0.98-1.00)	1.183(66.57)	749(65.88)	1.00(0.97–1.04)
On being referred urgently to secondary care. the patient does wait a long time to be seen		1.444(50.07)	1.114(54.58)	1.01(1.00-1.03)	886(82.42)	774(85.34)	1.04(0.99–1.08)	558(30.85)	340(29.98)	1.0(0.92–1.09)
On being sent back to primary care. the patient does wait a long time to be seen***		480(23.76)	542(34.05)	**1.52(1.26–1.83)**	193(18.16)	198(21.98)	**1.25(1.04–1.50)**	287(29.99)	344(49.78)	**1.72(1.66–1.79)**
***General perception of coordination across levels of care***										
I think that in this area patient care is coordinated between PC and SC doctors		1.012(34.43)	549(26.82)	**0.78 (0.66–0.93)**	339(32.13)	215(24.80)	0.82(0.62–1.09)	673(35.72)	334(28.31)	**0.78(0.69–0.88)**

^a^ Year of reference: 2017. ^b^ the value corresponds to the categories always/very often. ^c^ Adjusted by level of care, sex, years working as a doctor, type of specialty, type of hospital. ^d^ Adjusted by sex, years working as a doctor, type of specialty, type of hospital. Variable type of area according to the managing of PC/SC was used to adjust for a possible cluster effect. *** Percentage of Don’t Know /No Answer higher than 30%. Bold text indicates statistically significant associations

Regarding cross-level coordination of clinical management, when compared with 2017, there were improvements in the relative high levels of experience but also setbacks in 2022. Regarding consistency of care, there was an increase in the relatively high proportion of doctors who reported agreeing with the treatments prescribed at the other care level (PR:1.02, CI95% 1.01–1.04), which was higher in PC doctors (PR:1.09, CI95% 1.06–1.12). However, there was a worsening in the already very low proportion of doctors who reported that joint patient management plans were established when needed (PR:0.90, CI95% 0.83–0.97) with no differences between levels, and an increase in the repetition of tests (PR:1.19, CI95% 1.06–1.32) [Table 2].

Concerning adequate follow-up between levels, when compared with 2017, the high proportion of doctors who considered that patients were adequately referred to SC decreased, and this decrease was higher in SC doctors (PR:0.96, CI95% 0.92–0.99). Likewise, the relatively high proportion of PC doctors who reported that SC doctors send patients to PC when appropriate decreased (PR:0.94, CI95% 0.91–0.97). Although there is still room for improvement, the proportion of PC doctors who reported receiving follow-up recommendations from SC doctors improved (PR:1.16, CI95% 1.09–1.24) and the proportion of doctors of both levels that reported that PC doctors consult with SC doctors doubts on the patient follow-up also increased (PR:1.10, CI95% 1.05–1.15) and this increase was higher among PC doctors [Table 2].

Regarding accessibility between levels, there was an increase in the already high proportion of doctors at both levels who reported long waiting times for patients to be seen after being returned to PC (PR: 1.52, CI95% 1.26–1.83), which was higher for SC doctors [Table 2].

**Fig. 1:**
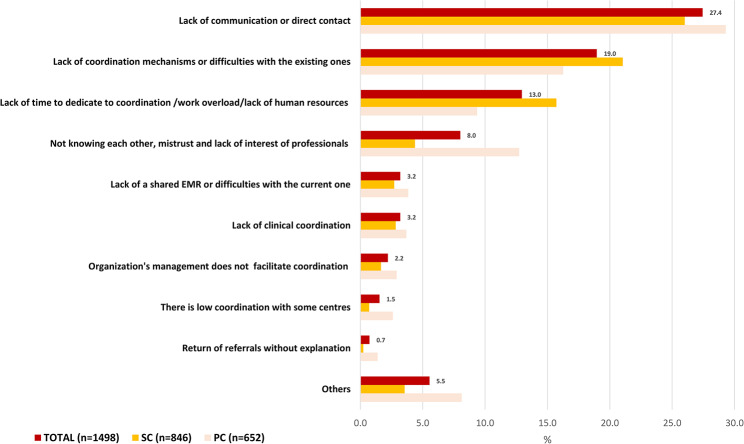
Reasons for a low perception of clinical coordination within the area, total and by level of care. Year 2022

**Fig. 2:**
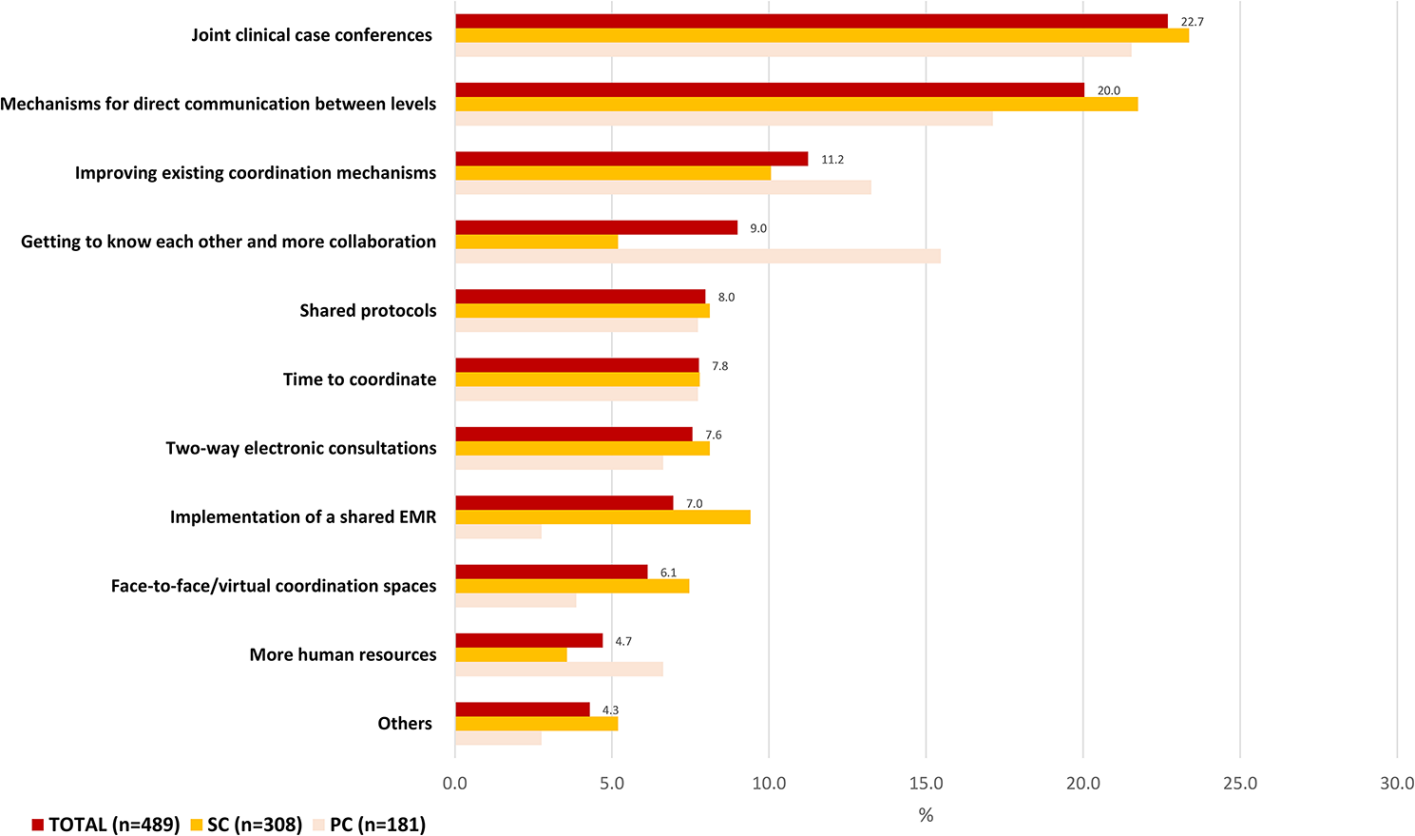
Suggestions for improving coordination, total and by level of care. Year 2022

Finally, the already low perception of cross-level clinical coordination in the healthcare are in 2017 has worsened by 2022 (PR: 0.78, CI95% 0.66–0.93) and more so among SC doctors (PR: 0.78, CI95% 0.69–0.88) [Table 2]. Reasons for considering the coordination to be low continued to be the limited direct communication between professionals and the insufficient availability of coordination mechanisms that promote cross-level communication [Fig. 1]. In the same line, main suggestions for improvement of cross-level clinical coordination included implementation of joint clinical case conferences and other mechanisms for direct communication between levels, as well as improving the existing coordination mechanisms [Fig. 2].

### Changes in factors influencing cross-level clinical coordination, including use of ICT-based coordination mechanisms

Compared with 2017, there was a worsening of some organisational factors that influence cross-level clinical coordination, with an improvement in use of some ICT-based coordination mechanisms in 2022. On the one hand, there was a decrease in the already relatively low proportion of doctors reporting that their organisation’s management facilitated cross-level clinical coordination (PR: 0.78, CI95% 0.75–0.81) or set objectives aimed at cross-level clinical coordination (PR: 0.82, CI95% 0.78–0.87) and this decrease was higher among SC doctors [Table 3]. The proportion of those reporting to have enough time to dedicate to coordination, which was already low in 2017, decrease even more in 2022, especially among SC doctors (PR: 0.70, CI95% 0.61–0.81) [Table 3].

**Table 3:** Changes in factors related to clinical coordination between 2017 and 2022, in total and, by level of care

		Total			Primary Care			Secondary care		
		2017 *N* = 3308	2022 *N* = 2277	2022/2017^a^ changes	2017 *N* = 3308	2022 *N* = 2277	2022/2017^a^ changes	2017 *N* = 3308	2022 *N* = 2277	2022/2017^a^ changes
		n (%)^*b*^	n (%)^*b*^	a PR (CI 95%) ^*c*^	n (%)^*b*^	n (%)^*b*^	a PR (CI 95%) ^*d*^	n (%)^*b*^	n (%)^*b*^	a PR (CI 95%) ^*d*^
***Organisational factors***										
My organisation’s management facilitates coordination between PC and SC doctors		1.492(59.18)	707(46.00)	**0.78(0.75–0.81)**	665(67.44)	412(55.83)	**0.84(0.81–0.87)**	827(53.88)	295(36.92)	**0.72 (0.66–0.79)**
My organisation sets objectives that are aimed at coordination between care levels		1.370(55.20)	660(44.75)	**0.82(0.78–0.87)**	540(56.72)	317(46.14)	**0.83(0.77–0.90)**	830(54.25)	343(43.53)	**0.82(0.79–0.86)**
The time I can dedicate to coordinating with doctors of the other level during my working day is sufficient		380(13.90)	180(10.24)	**0.72(0.59–0.89)**	136(13.67)	82(10.69)	0.77(0.56–1.05)	244(14.03)	98(9.89)	**0.70(0.61–0.81)**
***Interactional factors***										
I know the doctors of the other care level who see my patients personally		1.103(39.06)	395(21.37)	**0.56(0.43–0.72)**	442(44.02)	180(23.17)	**0.53(0.48–0.60)**	661(36.32)	215(20.07)	**0.59(0.43–0.83)**
I trust in the clinical skills of the doctors of the other level who see my patients		2.429(88.62)	1.547(88.15)	0.97(0.92–1.02)	971(97.20)	733(95.94)	0.99(0.94–1.04)	1.458(83.70)	814(82.14)	0.95(0.90–1.08)
My daily practice influences the practice of the doctors of the other level		1.834(78.61)	1.176(79.73)	1.04(0.99–1.11)	554(66.19)	450(71.20)	1.12(0.99–1.26)	1.280(85.56)	726(86.12)	1.02(0.98–1.06)
In practice. primary care doctors are responsible for coordinating the patients on their way through the different care levels		2.189(81.92)	1.432(84.88)	**1.03(1.01–1.04)**	950(95.19)	706(92.77)	0.98(0.96–1.01)	1.239(74.01)	726(78.40)	**1.07(1.03–1.10)**
***Attitudinal factors***										
Satisfaction with the job in the organisation ^*d*^		2.193(75.70)	1.262(66.91)	**0.91(0.89–0.93)**	741(74.17)	533(68.25)	**0.89(0.85–0.94)**	1.452(76.50)	729(65.97)	**0.91(0.85–0.97)**
***Frequent Use of ICT-based coordination mechanisms*** ^e^										
Shared EMR of Catalonia (HC3/HES) ^*f*^		1.939(65.86)	1.027(78.88)	**1.21(1.15–1.27)**	802(77.26)	442(90.20)	1.17(1.13–1.21)	1.137(59.65)	585(72.04)	**1.28(1.12–1.46)**
Shared EMR of the centre ^*f*^		2.044(81.27)	1.171(88.51)	**1.07(1.04–1.11)**	836(94.04)	559(95.39)	1.01(0.97–1.05)	1.208(74.29)	612(83.04)	**1.14(1.10–1.20)**
Electronic consultations through the EMR^g^		854(52.17)	509(67.60)	**1.30 (1.20–1.39)**	430(52.96)	308(78.37)	**1.48(1.29–1.70)**	424(51.39)	201(55.83)	**1.07(1.02–1.13)**
Consultations through e-mail^g^		460(26.11)	139(17.25)	**0.66(0.50–0.88)**	179(23.31)	59(15.40)	**0.61(0.43–0.84)**	281(28.27)	80(18.91)	0.67(0.45–1.02)
Consultations through telephone^g^		425(21.73)	81(20.00)	0.94(0.86–1.03)	86(12.80)	21(13.29)	1.08(0.81–1.44)	339(26.40)	60(24.29)	0.99(0.93–1.05)
Joint clinical case conferences^h^		748(62.49)	167(57.79)	**0.89(0.81–0.98)**	473(69.46)	80(51.61)	**0.75(0.61–0.92)**	275(53.29)	87(64.93)	1.14(0.98–1.30)

^a^ Year of reference: 2017

^b^ the value corresponds to the categories always/very often

^c^ Adjusted by level of care, sex, years working as a doctor, type of specialty, type of hospital

^d^ the value corresponds to the category: yes

^e^ Adjusted by sex, years working as a doctor, type of specialty, type of hospital

The value corresponds to the category frequent use: ^f^ Daily, ^g^ Daily or Weekly and, ^h^ Daily, Weekly or Monthly within those who reported to have access [Additional file 4]

Variable type of area according to the managing of PC/SC was used to adjust for a possible cluster effect. Bold text indicates statistically significant associations

On the other hand, in 2022 there was an increase, among doctors from both levels of care who had access to [Additional file 4] and frequent use of some ICT-based coordination mechanisms such as, the shared EMR of the region (HC3/HES) (PR:1.21, CI95% 1.15–1.27) and the shared EMR of the organisations (PR:1.07, CI95% 1.04–1.11), that was already relatively high. This increase in use for both EMRs was higher among SC doctors, although they continue to be more used by PC doctors. There was also an increase in the relatively low proportion of doctors using electronic consultations between levels through EMR, especially by PC doctors (PR:1.48, CI95% 1.29–1.70) [Table 3]. Nonetheless, difficulties in the use of the mechanisms such as contradictory or disorganised information, technical problems, or lack of relevant information were reported [Additional file 5]. In addition, participation in joint clinical case conferences (PR: 0.89, CI95% 0.81–0.98) and use of email consultations (PR:0.66 CI95% 0.50–0.88) decreased, especially among PC doctors [Table 3].

As for interactional factors related to cross-level clinical coordination, when compared with 2017, the already low proportion of doctors that reported knowing doctors from the other level decreased (PR:0.56 CI95%. 0.43–0.72), especially among PC doctors. While factors such as trusting in the clinical skills of the other level’s doctors and perceiving that their own practice influences the other level’ remained high, with no differences between years or levels. Finally, doctors’ relatively high satisfaction with their job in their organisations fell significantly at both levels of care (PR: 0.91, CI95% 0.89–0.93) [Table 3].

## Discussion

Improving cross-level clinical coordination is essential for healthcare systems based on PC, given the increasingly complex health needs of the patients, which often require care by multiple professionals at different levels of care [3, 4]. To address this challenge in recent years European healthcare systems have promoted the implementation of ICT-based coordination mechanisms as tools to improve communication and collaboration between professionals, as well as accessibility, quality, and efficiency [37]. Moreover, the disruption generated by the COVID-19 pandemic to the health services accelerated the introduction of those mechanisms, among other measures, but their impact on clinical coordination and quality of care is unknown and needs to be analysed to guide future strategies and to contribute to increase health systems resilience. This is the first study that comprehensively analyse changes in the experience and perception of clinical coordination of PC and SC doctors, and related factors, in a NHS following the pandemic, allowing the identification of areas for improvement.

Results show, with some differences between levels, that in 2022 the experience of cross-level coordination of clinical information remained relatively high, with slight improvement, while the experience of coordination of clinical management showed both improvements and setbacks related to cross-level care consistency and patient follow-up. Cross-level accessibility continued to be low and, particularly access to PC has worsened. Likewise, the already low perception of clinical coordination in the healthcare area worsened. There were also setbacks in the organisational factors related to coordination, although there was an improvement in the use of some ICT-based coordination mechanisms. In addition, interactional and attitudinal factors worsened.

### Changes in the experience of clinical coordination highlights some resilience but also the need for improvement measures

Despite the relatively high experience of coordination of cross-level clinical information that remained in 2022, there was a worsening of some aspects related to consistency of care, such as tests repetition and contraindications and/or duplications of prescribed treatments, which should have improved with the increased use of the ICT-based coordination mechanisms (EMR, electronic consultations). This is probably related, on the one hand, to the difficulties reported such as, technical problems or outdated or contradictory information [38] and on the other hand, to the uneven implementation throughout the healthcare areas [39, 40] due to the diversity of service provision that characterises the Catalan NHS [52–54], pointing out the difficulties of implementing shared ICT-based coordination mechanisms and the efforts to overcome them [15, 25, 28]. Therefore, it is necessary for the health authority to address the problems related to the interoperability and uneven implementation of EMRs and electronic consultations [41, 42], promoting, in collaboration with the different stakeholders, the implementation of a single electronic health record throughout the territory, on which the rest of the ICT-based coordination mechanisms are based [43, 44].

Regarding clinical management coordination, there was an improvement in the agreement on prescribed treatments, consultation of doubts by PC and recommendations made by SC that could be related to the increase in the use of electronic consultations through the EMR, whose main use is to request the clinical opinion of colleagues in their area of expertise [11, 17]. However, it seems to be insufficient to improve the limited joint definition of individualized treatments plans, which fell even more in 2022. This could be explained by that shared management of patients with complex needs requires other types of direct synchronous feedback mechanisms (e.g., telephone consultations, joint clinical case conferences) [11, 43, 44]. The results of the study show precisely that the low mutual knowledge reported by doctors before the pandemic has been intensified, among others, because of the backlog in the use of mechanisms that allow direct contact such as joint clinical case conferences and the high staff turnover that healthcare organisations have faced during the pandemic [45], making interaction between professionals even more difficult.

The COVID-19 added pressure to healthcare professionals [8, 19] by increasing work overload with subsequent mental stress and exhaustion, especially in PC [46, 47], and may have contributed to the increase of inadequate referrals reported by PC and SC doctors. On the one hand, some studies have shown that it may be more difficult for mentally stressed and exhausted doctors to perform an adequate anamnesis [48, 49] and thus make proper referrals and, on the other, the increased use of ICT-based mechanisms may have exacerbated the stress [50, 51] and increased the unnecessary referrals to SC to release work overload [52]. Moreover, as some of the PC doctors reported that SC doctors refused referrals without explanation, the lack of proper feedback could lead to repeated unnecessary referrals. So, further research is needed to analyse more in-depth the factors related with this worsening, as well as, to promote strategies to improve the working environment of professionals, since it might have a negative impact on coordination and quality of care.

Finally, the results show an important decrease in the already limited accessibility between levels before the pandemic, with long waiting times to SC, and, particularly to PC. These results are in line with the available evidence [20, 21, 30, 31] that analyse the consequences of the measures introduced during the pandemic (elective procedures postponed, resources redirected to COVID-19 care, shift to telemedicine, etc.) [8, 20, 37, 53], that affected the functioning of health services already under pressure (and underfunded) due to the austerity measures introduced during the last financial crisis [16]. These results call into question the effectiveness of measures that were put in place to improve accessibility during the pandemic, such as the use of electronic consultations between levels [16]. In this regard, some studies have linked their use to increased barriers of access to SC, as SC doctors could refuse face-to-face referrals until additional tests were performed, among others [54]. It is also necessary to strengthen access to PC, among others, by promoting reorganisation plans in PC that includes mixed face-to-face/telematic consultations and optimisation of resources, so that it can properly act as the gatekeeper to the NHS and coordinator of patients care throughout the healthcare process [8].

### Reduction in the poor perception of cross-level clinical coordination may be related to setbacks in organisational, interactional, and attitudinal factors during and after the pandemic

Despite maintenance or improvement in some aspects of the experience of clinical coordination between levels, there was a significant worsening in the perception of coordination in the healthcare area, already low before the pandemic, especially among SC doctors. This is congruent with a survey carried out with PC doctors of Catalonia during the first waves of the pandemic that showed a perception of lack of coordination, especially with emergency rooms and hospital outpatient care [32]. The results of the analysis of changes in factors related to coordination show that this drop may be related to: (1) the decrease in the use of synchronous coordination mechanisms such as joint clinical case conferences that facilitate direct communication, collaboration, and mutual knowledge [55]; (2) the decrease in institutional support to provide the appropriate conditions for coordination (time, common objectives, etc.); (3) the decrease in job satisfaction, also described in the literature [32, 56], and in addition to the above-mentioned factors, to the general worsening of working conditions and the burnout to which they are subjected [56, 57], especially in primary care [32]. The worsening of all these factors, which would have been exacerbated during the first waves of the pandemic, does not seem to have been reversed in the subsequent phases. Therefore, it is necessary to further analyse the causes of the high job dissatisfaction of professionals and organisational factors such as institutional support, to promote the implementation of strategies for their improvement.

### Improving doctors’ mutual knowledge and existing coordination mechanisms: strategies proposed by doctors to improve cross-level coordination

Results on doctors’ suggestions to improve clinical coordination between levels referred to improving organisational and interactional factors and are consistent with previous results [58], highlighting the way forward and the relevance of involving professionals in the selection and design of interventions [59]. First, they suggested the implementation of mechanisms that promote direct communication and knowledge between professionals, essential factors to improve the experience and perception of coordination [60, 61]. Although ICT-based coordination mechanisms (e.g., shared EMR or electronic consultations) have been introduced to address this issue [28], due to their potential for improving transfer of clinical information and communication between professionals [17, 38], they need to be used in combination with others that allow verbal communication, feedback and standardisation of processes [40, 55, 58, 62] -specifically joint clinical case conferences, direct synchronous communication channels (telephone) between PC and SC and shared protocols-, even though they are more time consuming, they allow to establish common clear pathways of diagnosis and treatment and collaboration [40, 55]. Hence, the importance of facilitating an organisational environment that allows their proper use: time and an increase in needed resources [63].

Second, they suggested the improvement of the existing coordination mechanisms, in keeping with the evidence that difficulties -especially those affecting interoperability and safety- can discourage their use and limit the impact on clinical coordination [38, 42, 51]. Interventions for improving clinical coordination are often introduced but are not designed or evaluated in a participatory way [64], although the involvement of professionals in the process can be relevant to correct deficiencies or difficulties that may arise and generates greater acceptance, increasing its sustainability over time [65]. In short, results show the need for development of multicomponent strategies that include the participation of professionals in the identification of difficulties and design of mechanisms, since their involvement is key to adapt the strategies to the conditions and needs of each context to ensure that they can be properly implemented [59].

One of the potential limitations of this study is that there may have been a selection bias due to the self-administered nature of the questionnaire and the non-probabilistic sampling of the areas. Nevertheless, the characteristics of the sample were similar to the universe of doctors in the Catalan NHS [66–68] and the diversity of the health areas of the Catalan Health System was represented. Finally, a drop in participation in 2022, which may have been influenced by the critical time in which the survey was conducted, the beginning of the recovery after the increase in infections by the omicron variant of COVID-19 [69]. Nevertheless, the participation rate was within the expected range for an online survey [70].

## Conclusions

The COVID-19 pandemic was a challenging milestone that put a great pressure on health systems. It led to the rapid adoption of strategies to ensure healthcare to the population such as ICT-based coordination mechanisms. However, its impact on clinical coordination is unknow. This study analysed the changes in the different dimensions of cross-level clinical coordination and related factors that occurred in the Catalan NHS after the pandemic. Its results help to identify areas of improvement and make recommendations that are also useful for others NHS.

Even though there was an increased use of coordination mechanisms such as shared EMRs or electronic consultations and an improvement in some elements of coordination of clinical information and of clinical management probably related to a greater use of these mechanisms, other aspects of coordination such as the adequacy of referrals and accessibility between care levels have worsened, contrary to expected. Therefore, further evaluation of the impact of ICT-based coordination mechanisms on care coordination, and the barriers and facilitators associated to its use, is needed. Likewise, particular attention must be paid to other issues that worsened and are associated to the low perception of general coordination seen such as institutional support for coordination, knowledge between doctors, and job satisfaction, as well as to the contextual elements that give rise to them and that have intensified following the pandemic, such as increased waiting lists, work overload, high staff turnover and worse working conditions.

In consequence, decision makers and managers would have to prioritise participatory strategies that facilitate direct communication and knowledge between professionals and to foster an organisational climate that facilitates its implementation and sustainability, as well as to address the difficulties detected in the existing ones. It is also necessary to encourage support and protection policies for healthcare professionals and services that improve the working environment, promote cross-level clinical coordination, and guarantee quality and efficient care for more resilient NHS.

### Electronic supplementary material

Below is the link to the electronic supplementary material.

Supplementary Material 1

Supplementary Material 2

Supplementary Material 3

Supplementary Material 4

Supplementary Material 5

## Data Availability

No datasets were generated or analysed during the current study.

## Abbreviations

NHS: National Health System
PC: Primary Care
SC: Secondary Care
EMR: Electronic medical record

## References

[1] Ovretveit J. Does clinical coordination improve quality and save money - A summary review of the evidence. The health foundation [Internet]. 2011;2(June):1–30. http://www.ncbi.nlm.nih.gov/pubmed/21325659

[2] Motulsky A, Sicotte C, MP M, Schuster T, Girard N, Buckeridge D et al. Using Health Information Exchange: Usage and Perceived Usefulness in Primary Care. Stud Health Technol Inform [Internet]. 2019;264:709–13. https://pubmed.ncbi.nlm.nih.gov/31438016/10.3233/SHTI190315 31438016

[3] Sampson R, Cooper J, Barbour R, Polson R, Wilson P. Patients’ perspectives on the medical primary–secondary care interface: systematic review and synthesis of qualitative research. BMJ Open. 2015;5(10):e008708. 26474939 10.1136/bmjopen-2015-008708 PMC4611413

[4] Bywood P, Jackson-Bowers E, Muecke S. Initiatives to integrate primary and acute health care, including ambulatory care services. Adelaide: Primary Health Care Research & Information Service; 2011.

[5] Simpson K, Nham W, Thariath J, Schafer H, Greenwood-Eriksen M, Fetters MD et al. How health systems facilitate patient-centered care and care coordination: a case series analysis to identify best practices. BMC Health Serv Res [Internet]. 2022;22(1):1448. https://bmchealthservres.biomedcentral.com/articles/10.1186/s12913-022-08623-w 10.1186/s12913-022-08623-w PMC9710067 36447273

[6] Graetz I, Reed M, Shortell S, Rundall T, Bellows J, Hsu J et al. The next step towards making use meaningful: electronic information exchange and care coordination across clinicians and delivery sites. Med Care [Internet]. 2014;52(12):1037–41. https://pubmed.ncbi.nlm.nih.gov/25304020/10.1097/MLR.0000000000000245 PMC5131789 25304020

[7] Vimalananda VG, Orlander JD, Afable MK, Fincke BG, Solch AK, Rinne ST et al. Electronic consultations (E-consults) and their outcomes: a systematic review. Journal of the American Medical Informatics Association [Internet]. 2020;27(3):471–9. https://academic.oup.com/jamia/article/27/3/471/5588944 10.1093/jamia/ocz185 PMC7647247 31621847

[8] Satué de Velasco E, Gayol Fernández M, Eyaralar Riera MT, Magallón Botaya R, Abal Ferrer F. Impact of the pandemic on primary care. SESPAS Report 2022. Gac Sanit. 2022;36:S30–5. 35781145 10.1016/j.gaceta.2022.05.004 PMC9244614

[9] Mohammed HT, Hyseni L, Bui V, Gerritsen B, Fuller K, Sung J et al. Exploring the use and challenges of implementing virtual visits during COVID-19 in primary care and lessons for sustained use. Prazeres F, editor. PLoS One [Internet]. 2021;16(6):e0253665. 10.1371/journal.pone.0253665 10.1371/journal.pone.0253665 PMC8224904 34166441

[10] Longest B, Young G. Coordination and communication. In: Shortell S, Kaluzny A, editors. Healthcare Management: Organization Design and Behavior. 4th ed. New York: Delmar; 2000. pp. 210–43.

[11] Terraza R, Vargas I, Vázquez ML. La coordinación entre niveles asistenciales: una sistematización de sus instrumentos y medidas. Gac Sanit. 2006;20(6):485–95. 17198628 10.1157/13096516

[12] Reid R, Haggerty JM. Defusing the Confusion: Concepts and Measures of Continuity of Healthcare. [Internet]. Ottawa, Ontario; 2002. https://www.researchgate.net/publication/245856177_Defusing_the_Confusion_Concepts_and_Measures_of_Continuity_of_Health_Care

[13] Vázquez ML, Vargas I, Garcia-Subirats I, Unger JP, De Paepe P, Mogollón-Pérez AS et al. Doctors’ experience of coordination across care levels and associated factors. A cross-sectional study in public healthcare networks of six Latin American countries. Soc Sci Med [Internet]. 2017;182:10–9. https://linkinghub.elsevier.com/retrieve/pii/S0277953617302241 10.1016/j.socscimed.2017.04.001 28411523

[14] Pérez A. Retos, logros y dificultades del médico de familia ante la pandemia por COVID-19 en Andalucía. Actual Med [Internet]. 2021;106(814):Supl2: 109–117. https://actualidadmedica.es/articulo-suplementos/supl814-2_re15/

[15] Servei Català de la Salut. Generalitat de Catalunya. Mapa estratègic CatSalut 2022–2024. Reptes i full de ruta. 2022.

[16] Fahy N, Williams GA, Habicht T et al. Use of digital health tools in Europe: Before, during and after COVID-19 [Internet]. Copenhague; 2021 [cited 2022 Feb 10]. https://www.ncbi.nlm.nih.gov/books/NBK576970/pdf/Bookshelf_NBK576970.pdf 35099866

[17] Vimalananda VG, Gupte G, Seraj SM, Orlander J, Berlowitz D, Fincke BG, et al. Electronic consultations (e-consults) to improve access to specialty care: a systematic review and narrative synthesis. J Telemed Telecare. 2015;21(6):323–30. 25995331 10.1177/1357633X15582108 PMC4561452

[18] Jiménez Carrillo M, Martín Roncero U, Aldasoro Unamuno E, Morteruel Arizcuren M, Baza Bueno M. Percepciones y experiencias de la población ante la transformación de la modalidad de las consultas en atención primaria durante la pandemia. Aten Primaria. 2022;54(4):102263. 35144184 10.1016/j.aprim.2021.102263 PMC8841613

[19] Ares-Blanco S, Astier-Peña MP, Gómez-Bravo R, Fernández-García M, Bueno-Ortiz JM. The role of primary care during COVID-19 pandemic: a European overview. Aten Primaria. 2021;53(8). 10.1016/j.aprim.2021.102134 PMC8196323 34237607

[20] van Ginneken E, Reed S, Siciliani L, Eriksen A, Schlepper L, Tille F et al. Addressing backlogs and managing waiting lists during and beyond the COVID-19 pandemic. 2022. 36800878

[21] NHS Staff Survey Co-ordination Centre. NHS Staff Survey National Results. London; 2021.

[22] House S, Crandell J, Stucky C, Kitzmiller R, Jones C, Gittell JH. Relational Coordination as a Predictor of Job Satisfaction and Intent to Stay Among Nurses and Physicians in the Military Health System. Mil Med [Internet]. 2023;188(1–2):e316–25. https://academic.oup.com/milmed/article/188/1-2/e316/6432365 10.1093/milmed/usab464 35050374

[23] Capone V, Borrelli R, Marino L, Schettino G. Mental Well-Being and Job Satisfaction of Hospital Physicians during COVID-19: Relationships with Efficacy Beliefs, Organizational Support, and Organizational Non-Technical Skills. Int J Environ Res Public Health [Internet]. 2022;19(6):3734. https://www.mdpi.com/1660-4601/19/6/3734 10.3390/ijerph19063734 PMC8948767 35329420

[24] Bernal-Delgado E, Garcia-Armesto S, Oliva J, Sanchez Martinez FI, Repullo JR, Pena-Longobardo LM et al. Spain: Health System Review. Health Syst Transit [Internet]. 2018;20(2):1–179. http://www.ncbi.nlm.nih.gov/pubmed/30277216 30277216

[25] Direcció General de Planifcació en Salut. Pla de salut de Catalunya 2021–2025 [Internet]. Barcelona, Spain. 2021. https://hdl.handle.net/11351/7948

[26] Esteve-Matalí L, Vargas I, Cots F, Ramon I, Sánchez E, Escosa A, et al. Does the integration of health services management improve clinical coordination? Experience in Catalonia. Gac Sanit. 2022;36(4):324–32. 34334227 10.1016/j.gaceta.2021.06.004

[27] Vargas I, Vázquez ML, Henao D, Calpe JF. De la competència a la col·laboració. Experiència en la integració assistencial a Catalunya. Fulls econòmics del sistema sanitari [Internet]. 2009;38:27. https://dialnet.unirioja.es/servlet/articulo?codigo=3492040

[28] Generalitat de Catalunya. Departament de salut. The Catalan Information Systems Master Plan: Building a digital health strategy for Catalonia together [Internet]. 2017. https://salutweb.gencat.cat/web/.content/_ambits-actuacio/Linies-dactuacio/tic/pdsis/pdsis-en.pdf

[29] Vazquez ML, Amigo F, Esteve-Matalí L, Vargas I. La coordinación clínica entre niveles de atención: Resultados comparativos entre áreas del sistema sanitario en Cataluña [Internet]. Barcelona, Spain; 2021. http://www.consorci.org/media/upload/arxius/coneixement/COORDENA.CAT/cast Informe general Coordena CAT_final2.pdf.

[30] García-Rojo E, Manfredi C, Santos-Pérez-de-la-Blanca R, Tejido-Sánchez, García-Gómez B, Aliaga-Benítez M, et al. Impact of COVID-19 outbreak on urology surgical waiting lists and waiting lists prioritization strategies in the Post-COVID-19 era. Actas Urol Esp. 2021;45(3):207–14. 33546905 10.1016/j.acuro.2020.11.001

[31] Pavón I, Rosado Sierra J, Salguero Ropero A, Viedma Torres V, Guijarro de Armas G, Cuesta Rodríguez-Torices M et al. [E-consultation as a tool for the relationship between Primary Care and Endocrinology. Impact of COVID-19 epidemic in its use]. J Healthc Qual Res [Internet]. 2022;37(3):155–61. https://pubmed.ncbi.nlm.nih.gov/34866028/10.1016/j.jhqr.2021.10.006 PMC9847372 34866028

[32] Societat Catalana de Medicina Familiar i Comunitària. Atenció Primària en l’era post-COVID: revolució per a la transformació. Barcelona; 2021.

[33] Muñoz-Cobo Orosa B, Pérez García M, Rodríguez Ledott M, Varela Serrano C, Sanz Valero J. Satisfacción laboral y calidad de vida de Los médicos residentes españoles durante la pandemia por la COVID-19. Med Segur Trab (Madr). 2022;67(264):169–90.

[34] Campaz D, Vargas I, Plaja P, Sanclemente M, Paino M, Madrid M et al. A questionnaire to measure the impact of ICT-based coordination mechanisms on clinical coordination. Eur J Public Health [Internet]. 2022;32(Supplement_3). https://academic.oup.com/eurpub/article/doi/10.1093/eurpub/ckac130.118/6765902

[35] Vazquez ML, Vargas I, Unger JP, De Paepe P, Mogollon-Perez AS, Samico I, et al. Evaluating the effectiveness of care integration strategies in different healthcare systems in Latin America: the EQUITY-LA II quasi-experimental study protocol. BMJ Open. 2015;5(7):e007037. 26231753 10.1136/bmjopen-2014-007037 PMC4521516

[36] Vázquez ML, Vargas I, Romero A, Sánchez E, Ramon I, Plaja P, et al. Adapting the COORDENA questionnaire for measuring clinical coordination across health care levels in the public health system of Catalonia (Spain). Public Health Panorama. 2018;4(4):491–735.

[37] Fahy N, Williams GA, Habicht T, et al. Use of digital health tools in Europe: before, during and after COVID-19. Copenhague; 2021. 35099866

[38] Motulsky A, Sicotte C, MP M, Schuster T, Girard N, Buckeridge D, et al. Using Health Information Exchange: usage and Perceived Usefulness in Primary Care. Stud Health Technol Inf. 2019;264:709–13. 10.3233/SHTI190315 31438016

[39] Schoen C, Osborn R, Squires D, Doty M, Rasmussen P, Pierson R et al. A Survey Of Primary Care Doctors In Ten Countries Shows Progress In Use Of Health Information Technology, Less In Other Areas. Health Aff [Internet]. 2012;31(12):2805–16. http://www.healthaffairs.org/doi/10.1377/hlthaff.2012.0884 10.1377/hlthaff.2012.0884 23154997

[40] Aller MB, Vargas I, Coderch J, Vázquez ML. Doctors’ opinion on the contribution of coordination mechanisms to improving clinical coordination between primary and outpatient secondary care in the Catalan national health system. BMC Health Serv Res. 2017;17(1):1–11. 29273045 10.1186/s12913-017-2690-5 PMC5741878

[41] Li E, Clarke J, Ashrafian H, Darzi A, Neves AL. The Impact of Electronic Health Record Interoperability on Safety and Quality of Care in High-Income countries: systematic review. J Med Internet Res. 2022;24(9):e38144. 36107486 10.2196/38144 PMC9523524

[42] Dutta B, Hwang HG. The adoption of electronic medical record by physicians. Medicine. 2020;99(8):e19290. 32080145 10.1097/MD.0000000000019290 PMC7034652

[43] Secretaria General de Salud Digital Información e Innovación para el SNS. Estrategia de salud digital: Sistema Nacional de Salud [Internet]. 2021. https://www.sanidad.gob.es/ciudadanos/pdf/Estrategia_de_Salud_Digital_del_SNS.pdf

[44] Miloris K, Papageorgiou K. A Study on Healthcare ICT Systems and their Usefulness During CoVid-19 Focused in the European Environment. Journal of Hospital and Healthcare Administration [Internet]. 2021;5(1). https://www.gavinpublishers.com/article/view/a-study-of-healthcare-ict-systems-and-their-usefulness-during-covid19-focused-in-the-european-environment-1

[45] Randstad Research. Informe de rotación laboral en España 2022. 2022. Available on: https://www.randstadresearch.es/informe-rotacion-2022/

[46] Seda-Gombau G, Montero-Alía JJ, Moreno-Gabriel E, Torán-Monserrat P. Impact of the COVID-19 pandemic on Burnout in Primary Care Physicians in Catalonia. Int J Environ Res Public Health. 2021;18(17):9031. 34501618 10.3390/ijerph18179031 PMC8431168

[47] Prasad K, McLoughlin C, Stillman M, Poplau S, Goelz E, Taylor S, et al. Prevalence and correlates of stress and burnout among U.S. healthcare workers during the COVID-19 pandemic: a national cross-sectional survey study. EClinicalMedicine. 2021;35:100879. 34041456 10.1016/j.eclinm.2021.100879 PMC8141518

[48] Kushnir T, Greenberg D, Madjar N, Hadari I, Yermiahu Y, Bachner YG. Is burnout associated with referral rates among primary care physicians in community clinics? Fam Pract. 2014;31(1):44–50. 24148815 10.1093/fampra/cmt060 PMC5926437

[49] Mangory KY, Ali LY, Rø KI, Tyssen R. Effect of burnout among physicians on observed adverse patient outcomes: a literature review. BMC Health Serv Res. 2021;21(1):369. 33879135 10.1186/s12913-021-06371-x PMC8057942

[50] Bahr TJ, Ginsburg S, Wright JG, Shachak A. Technostress as source of physician burnout: an exploration of the associations between technology usage and physician burnout. Int J Med Inf. 2023;177:105147. 10.1016/j.ijmedinf.2023.105147 37517300

[51] Osman MA, Schick-Makaroff K, Thompson S, Bialy L, Featherstone R, Kurzawa J, et al. Barriers and facilitators for implementation of electronic consultations (eConsult) to enhance access to specialist care: a scoping review. BMJ Glob Health. 2019;4(5):1–16. 10.1136/bmjgh-2019-001629 PMC6747903 31565409

[52] Gupte G, Vimalananda V, Simon SR, DeVito K, Clark J et al. Disruptive innovation: implementation of electronic consultations in a veterans affairs health care system. JMIR Med Inf. 2016;4(1):e6. 10.2196/medinform.4801 PMC4769358 26872820

[53] Pujolar G, Oliver-Anglès A, Vargas I, Vázquez ML. Changes in Access to Health Services during the COVID-19 pandemic: a scoping review. Int J Environ Res Public Health. 2022;19(3):1749. 35162772 10.3390/ijerph19031749 PMC8834942

[54] Lee MS, Ray KN, Mehrotra A, Giboney P, Yee HF, Barnett ML. Primary care practitioners’ perceptions of electronic Consult systems. JAMA Intern Med. 2018;178(6):782. 29801079 10.1001/jamainternmed.2018.0738 PMC6145753

[55] Henao D, Vázquez ML, Vargas I. Factores que influyen en la coordinación entre niveles asistenciales según la opinión de directivos y profesionales sanitarios. Gac Sanit. 2009;23(4):280–6. 19250716 10.1016/j.gaceta.2008.05.001

[56] Alrawashdeh HM, Al-Tammemi AB, Alzawahreh MKh, Al-Tamimi A, Elkholy M, Al Sarireh F, et al. Occupational burnout and job satisfaction among physicians in times of COVID-19 crisis: a convergent parallel mixed-method study. BMC Public Health. 2021;21(1):811. 33906619 10.1186/s12889-021-10897-4 PMC8079229

[57] Costello H, Walsh S, Cooper C, Livingston G. A systematic review and meta-analysis of the prevalence and associations of stress and burnout among staff in long-term care facilities for people with dementia. Int Psychogeriatr. 2019;31(08):1203–16. 30421691 10.1017/S1041610218001606

[58] Esteve-Matalí L, Vargas I, Amigo F, Plaja P, Cots F, Mayer EF, et al. Understanding how to improve the use of clinical coordination mechanisms between primary and secondary care doctors: clues from Catalonia. Int J Environ Res Public Health. 2021;18(6):1–18. 10.3390/ijerph18063224 PMC8003988 33804691

[59] Colquhoun HL, Squires JE, Kolehmainen N, Fraser C, Grimshaw JM. Methods for designing interventions to change healthcare professionals’ behaviour: a systematic review. Implement Sci. 2017;12(1):1–11. 28259168 10.1186/s13012-017-0560-5 PMC5336662

[60] Aller MB, Vargas I, Coderch J, Calero S, Cots F, Abizanda M et al. Doctors’ opinions on clinical coordination between primary and secondary care in the Catalan healthcare system. Gac Sanit. 2019. 10.1016/j.gaceta.2017.06.001 28844783

[61] Esteve-Matalí L, Vargas I, Sánchez E, Ramon I, Plaja P, Vázquez ML. Do primary and secondary care doctors have a different experience and perception of cross-level clinical coordination? Results of a cross-sectional study in the catalan national health system (Spain). BMC Fam Pract. 2020;21(1). 10.1186/s12875-020-01207-9 PMC7346358 32640991

[62] Collins SA, Bakken S, Vawdrey DK, Coiera E, Currie L. Clinician preferences for verbal communication compared to EHR documentation in the ICU. Appl Clin Inf. 2011;02(02):190–201. 10.4338/ACI-2011-02-RA-0011 PMC3631921 23616870

[63] Mitchell GK, Del Mar CB, Clavarino AM, Jong IC, Kennedy R. General practitioner attitudes to case conferences: how can we increase participation and effectiveness? Medical Journal of Australia. 2002;177(2):95–7. 10.5694/j.1326-5377.2002.tb04680.x 12098350

[64] Tang T, Lim ME, Mansfield E, McLachlan A, Quan SD. Clinician user involvement in the real world: Designing an electronic tool to improve interprofessional communication and collaboration in a hospital setting. Int J Med Inf. 2018;110:90–7. 10.1016/j.ijmedinf.2017.11.011 29331258

[65] Loewenson R, Laurell AC, Hogstedt C, D’Ambruoso L, Shroff Z. Participatory action research in health systems: a methods reader. AHPSR, WHO, IDRC Canada, Equinet: TARSC; 2014.

[66] Solsona i Pairó M, Treviño R, Merino M, Ferrer L. Demografia de les Professions Sanitàries a Catalunya: Anàlisi dels Estocs Actuals de Professionals Sanitari. Barcelona, Spain; 2006.

[67] Instituto de estadística de Cataluña. Personal hospitalario. Por categorias, tipos de concierto y tipos de hospital Cataluña. Internet. 2017;2019(cited 2024 Mar 5):Availablefromhttpswwwidescatcatindicadors–idaecn15813.

[68] Instituto nacional de estadística. N^o^ de Médicos por Comunidades, Ciudades autónomas y Provincias de colegiación, edad y sexo. [Internet]. 2022 [cited 2023 Oct 13]. https://www.ine.es/jaxi/Tabla.htm?tpx=59167&L=0

[69] Cunningham CT, Quan H, Hemmelgarn B, Noseworthy T, Beck CA, Dixon E, et al. Exploring physician specialist response rates to web-based surveys. BMC Med Res Methodol. 2015;15(1):32. 25888346 10.1186/s12874-015-0016-z PMC4404667

[70] Smyth J, Pearson J. Internet survey methods: a review of strengths, weaknesses, and innovations. In: Das M, Este P, Kaczmirek L, editors. Social and behavioral research and the internet advances in applied methods and research strategies. New York: Taylor and Francis Group; 2011. pp. 11–43.

