# Supplementary material for: Has cross-level clinical coordination changed in the context of the pandemic? The case of the Catalan health system
Source: BMC Health Serv Res. 2024 Aug 21;24:959. doi: 10.1186/s12913-024-11445-7 (PMC11337784; doi:10.1186/s12913-024-11445-7)
Supplement: Supplementary file 1 — Supplementary Material 1 [file 12913_2024_11445_MOESM1_ESM.docx]

# Additional File 1. Links to the COORDENA-CAT (2017) and COORDENA-TICs (2022) questionnaires

1. [Qüestionari COORDENA-CAT | Catàleg de publicacions | Consorci de Salut i Social de Catalunya](https://www.consorci.org/coneixement/cataleg-de-publicacions/164/questionari-coordena-cat)
   1. English version available: [COORDENA CAT Questionnaire_EN.pdf (consorci.org)](https://www.consorci.org/media/upload/arxius/publicacions/questionari_COORDENA/COORDENA%20CAT%20Questionnaire_EN.pdf)
2. [Qüestionari COORDENA.TICs | Catàleg de publicacions | Consorci de Salut i Social de Catalunya](https://www.consorci.org/coneixement/cataleg-de-publicacions/203/questionari-coordenatics)
   1. Catalan version: [Cat_Cuestionario COORDENA.TICs_castellano_vf.pdf (consorci.org)](https://www.consorci.org/media/upload/arxius/coneixement/COORDENA.TICs/Q%C3%BCestionaris/Cat_Cuestionario%20COORDENA.TICs_castellano_vf.pdf)
