# Supplementary material for: Has cross-level clinical coordination changed in the context of the pandemic? The case of the Catalan health system
Source: BMC Health Serv Res. 2024 Aug 21;24:959. doi: 10.1186/s12913-024-11445-7 (PMC11337784; doi:10.1186/s12913-024-11445-7)
Supplement: Supplementary file 3 — Supplementary Material 3 [file 12913_2024_11445_MOESM3_ESM.docx]

# Additional File 4. Differences in access and frequency of use of coordination mechanisms between years, by level of care

| \|  \|  \| **Total** \| \| \| **Primary care** \| \| \| **Secondary care** \| \| \| \| --- \| --- \| --- \| --- \| --- \| --- \| --- \| --- \| --- \| --- \| --- \| \|  \|  \| **2017** \| **2022** \| **2022/2017** ^a^ **changes** \| **2017** \| **2022** \| **2022/2017** ^a^ **changes** \| **2017** \| **2022** \| **2022/2017** ^a^ **changes** \| \|  \|  \| **N (%)** \| **N (%)** \| a PR (CI 95%) ^f^ \| **N (%)** \| **N (%)** \| a PR (CI 95%) *^g^* \| **N (%)** \| **N (%)** \| a PR (CI 95%) *^g^* \|   **Supplementary table 1. Differences in access and frequency of use of coordination mechanisms between years, by level of care** | | | | | | | | | | |
| --- | --- | --- | --- | --- | --- | --- | --- | --- | --- | --- | --- | --- | --- | --- | --- | --- | --- | --- | --- | --- | --- | --- | --- | --- | --- | --- | --- | --- | --- | --- | --- | --- | --- | --- | --- | --- | --- | --- | --- | --- | --- | --- | --- |
| ***Shared EMR of Catalonia (HC3/HES*** | | | | | | | | | | |
|  |  | N=3067 | N=1347 |  | N=1072 | N=509 |  | N=1995 | N=838 |  |
| Have access ^b^ |  | 3.057(99.67) | 1.335(99.48) | 1.00(0.99-1.00) | 1.068(99.63) | 504(99.41) | 1.00(0.99-1.00) | 1.989(99.70) | 831(99.52) | 1.00(0.99-1.00) |
| Frequent use ^c^ |  | 1.939(65.86) | 1.027(78.88) | **1.21(1.15-1.27)** | 802(77.26) | 442(90.20) | **1.17(1.13-1.21)** | 1.137(59.65) | 585(72.04) | **1.28(1.12-1.46)** |
| ***Shared EMR of the centre*** | | | | | | | | | | |
|  |  | N=2917 | N=1698 |  | N=1048 | N=671 |  | N=1869 | N=1027 |  |
| Have access ^b^ |  | 2.623(89.92) | 1.352(90.62) | 0.99(0.96-1.03) | 918(87.60) | 591(93.22) | 1.06(0.99-1.14) | 1.705(91.23) | 761(88.69) | 0.95(0.89-1.01) |
| Frequent use ^c^ |  | 2.044(81.27) | 1.171(88.51) | **1.07(1.04-1.11)** | 836(94.04) | 559(95.39) | 1.01(0.97-1.05) | 1.208(74.29) | 612(83.04) | **1.14(1.10-1.20)** |
| ***Electronic consultations through the EMR*** | | | | | | | | | | |
|  |  | N=2474 | N=1562 |  | N=1011 | N=537 |  | N=1463 | N=1025 |  |
| Have access ^b^ |  | 1.795(72.55) | 782(67.12) | 0.93(0.85-1.03) | 841(83.18) | 397(81.86) | 0.98(0.94-1.01) | 954(65.21) | 385(56.62) | 0.92(0.80-1.06) |
| Frequent use ^d^ |  | 854(52.17) | 509(67.60) | **1.30 (1.20-1.39)** | 430(52.96) | 308(78.37) | **1.48(1.29-1.70)** | 424(51.39) | 201(55.83) | **1.07(1.02-1.13**) |
| ***Consultations through e-mail*** | | | | | | | | | | |
|  |  | N=2481 | N=1931 |  | N=996 | N=793 |  | N=1485 | N=1138 |  |
| Have access ^b^ |  | 1.885(75.98) | 836(56.79) | **0.77(0.75-0.79)** | 792(79.52) | 393(55.59) | **0.70(0.64-0.76)** | 1.093(73.60) | 443(57.91) | **0.82(0.77-0.88)** |
| Frequent use ^d^ |  | 460(26.11) | 139(17.25) | **0.66(0.50-0.88)** | 179(23.31) | 59(15.40) | **0.61(0.43-0.84)** | 281(28.27) | 80(18.91) | 0.67(0.45-1.02) |
| ***Consultations through telephone*** | | | | | | | | | | |
|  |  | N=2615 | N=1798 |  | N=976 | N=716 |  | N=1639 | N=1082 |  |
| Have access ^b^ |  | 2.085(79.73) | 425(32.12) | **0.43(0.40-0.45)** | 700(71.72) | 160(25.60) | **0.37(0.32-0.45)** | 1.385(84.50) | 265(37.97) | **0.46(0.41-0.52)** |
| Frequent use ^d^ |  | 425(21.73) | 81(20.00) | 0.94(0.86-1.03) | 86(12.80) | 21(13.29) | 1.08(0.81-1.44) | 339(26.40) | 60(24.29) | 0.99(0.93-1.05) |
| ***Joint clinical case conferences*** | | | | | | | | | | |
|  |  | N=2817 | N=1891 |  | N=1045 | N=754 |  | N=1772 | N=1137 |  |
| Have access ^b^ |  | 1.226(43.52) | 298(17.89) | **0.38(0.33-0.44)** | 696(66.60) | 160(21.92) | **0.32(0.26-0.40)** | 530(29.91) | 138(14.74) | **0.49(0.47-0.51)** |
| Frequent use ^e^ |  | 748(62.49) | 167(57.79) | **0.89(0.81-0.98)** | 473(69.46) | 80(51.61) | **0.75(0.61-0.92)** | 275(53.29) | 87(64.93) | 1.14(0.98-1.30) |

^a^ Year of reference: 2017.

^b^ the value corresponds to the category *Yes.*

The value corresponds to the category frequent use*: ^c^Daily, ^d^Daily or Weekly and, ^e^ Daily, Weekly or Monthly* within those who reported to have access.

^f^Adjusted by sex, level of care, years of experience as a doctor, type of specialty, type of hospital*.*

*^g^* Adjusted by sex, years of experience as a doctor, type of specialty, type of hospital.

Variable type of area was used to adjust for a possible cluster effect.
