# Supplementary material for: Has cross-level clinical coordination changed in the context of the pandemic? The case of the Catalan health system
Source: BMC Health Serv Res. 2024 Aug 21;24:959. doi: 10.1186/s12913-024-11445-7 (PMC11337784; doi:10.1186/s12913-024-11445-7)
Supplement: Supplementary file 5 — Supplementary Material 5 [file 12913_2024_11445_MOESM5_ESM.docx]

# Additional file 3. Analysis of the data including the participants who answered the categories do not know/no answer.

| **Supplementary table 1. Differences between the experience of clinical information and clinical management and general perception of clinical coordination between years, by level of care** | | | | | | | | | | |
| --- | --- | --- | --- | --- | --- | --- | --- | --- | --- | --- |
|  | | **Total** | | | **Primary care** | | | **Secondary care** | | |
|  | | **2017**  **N=3308** | **2022**  **N=2277** | **2022/2017** ^a^ **changes** | **2017**  **N=1141** | **2022**  **N=945** | **2022/2017** ^a^ **changes** | **2017**  **N=2167** | **2022**  **N=1332** | **2022/2017** ^a^ **changes** |
|  | | **n (%)** *^b^* | **n (%)** *^b^* | a PR (CI 95%) *^c^* | **n (%)** *^b^* | **n (%)** *^b^* | a PR (CI 95%) *^d^* | **n (%)** *^b^* | **n (%)** *^b^* | a PR (CI 95%) *^d^* |
| ***Coordination of clinical information between levels of care*** | | | | | | | | | | |
| PC and SC doctors share information on the care of patients we have in common |  | 1,959(62.41) | 1,520(67.20) | 1.07(0.95-1.20) | 757(69.96) | 694(74.38) | 1.09(0.94-1.28) | 1,202(58.43) | 826(62.15) | 1.07 (0.94-1.22) |
| The information we share is as required for the care of these patients |  | 2,224(70.72) | 1,755(77.59) | **1.07(1.04-1.10)** | 830(76.92) | 765(81.82) | **1.07 (1.03-1.11)** | 1,394(69.56) | 990(76.10) | **1.07 (1.03-1.13)** |
| PC and SC doctors use the information that we share |  | 2,489(79.39) | 1,822(80.62) | 0.99 (0.98-1.01) | 911(84.20) | 793(84.54) | 0.98(0.95-1.01) | 1,578(76.86) | 1,029(77.84) | 1.01(0.98 -1.05) |
| ***Coordination of clinical management: Care consistency between levels of care*** | | | | | | | | | | |
| We agree with the treatments prescribed or directions given to the patients by doctors of the other level |  | 2,357(75.06) | 1,757(77.61) | 1.02(1.00-1.03) | 792(73.00) | 743(78.96) | **1.09(1.06-1.13)** | 1,565(76.16) | 1,014(76.64) | 0.98(0.95-1.02) |
| There are contraindications and/or duplications in the treatments prescribed by primary and secondary care doctors |  | 920(29.28) | 701(30.96) | 1.02(0.97-1.08) | 416(38.52) | 370(39.36) | 0.99(0.89-1.10) | 504(24.44) | 331(25.00) | 1.10(1.02-1.18) |
| PC and SC doctors establish a treatment plan together for patients that require this |  | 422(13.44) | 269(11.92) | **0.89(0.82-0.98)** | 129(11.91) | 101(10.80) | 0.93(0.86-1.00) | 293(14.24) | 168(12.72) | 0.90(0.76-1.07) |
| We repeat the tests that doctors have already carried out at the other level |  | 872(27.73) | 733(32.32) | **1.18(1.06-1.33**) | 299(27.61) | 335(35.64) | **1.36(1.10-1.68)** | 573(27.79) | 398(29.97) | **1.10(1.08-1.12)** |
| ***Coordination of clinical management: Adequate follow up between levels of care*** | | | | | | | | | | |
| PC doctors refer the patients to secondary care when appropriate |  | 2,493(80.60) | 1,786(81.89) | **0.98(0.96-0.99)** | 1,060(98.24) | 901(98.79) | 1.00(0.99-1.00) | 1,433(71.15) | 885(69.74) | **0.95(0.93-0.98)** |
| SC doctors send the patients back to primary care for follow-up when appropriate |  | 2,484(80.52) | 1,700(78.05) | 0.97(0.95-1.00) | 811(75.30) | 655(71.66) | **0.95(0.92-0.98)** | 1,673(83.32) | 1,045(82.67) | 0.98(0.93-1.04) |
| SC doctors make recommendations to the primary care doctor on the follow-up of patients (diagnosis, treatment, other guidelines) |  | 1,780(57.68) | 1,287(59.09) | **1.05(1.03-1.08)** | 421(39.13) | 443(48.63) | **1.16(1.09-1.24)** | 1,359(67.61) | 844(66.61) | 1.01(0.99-1.05) |
| PC doctors clarify any doubts on the follow-up of patients with the secondary care doctors |  | 1,249(40.43) | 984(45.22) | **1.11(1.05-1.18)** | 588(54.49) | 572(62.79) | **1.20(1.09-1.33)** | 661(32.89) | 412(32.57) | **1.07(1.05-1.10)** |
| ***Coordination of clinical management: Accessibility between levels of care*** | | | | | | | | | | |
| On being referred in the normal way to secondary care, the patient does wait a long time to be seen |  | 2,240(72.70) | 1,639(75.22) | 1.01(0.99-1.04) | 1,057(98.14) | 890(97.27) | **0.99(0.98-0.99)** | 1,183(59.03) | 749(59.26) | 1.03(1.00-1.04) |
| On being referred urgently to secondary care, the patient does wait a long time to be seen |  | 1,444(46.82) | 1,114(51.31) | **1.01(1.01-1.02)** | 886(82.04) | 774(85.34) | 1.03(0.99-1.07) | 558(30.85) | 340(29.98)0. | 1.01(0.93-1.10) |
| On being sent back to primary care, the patient does wait a long time to be seen*** |  | 480(15.65) | 542(24.89) | **1.63(1.30-2.05)** | 193(17.95) | 198(21.71) | **1.24(1.03-1.57)** | 287(14.40) | 344(27.17) | **1.96(1.80-2.13)** |
| ***General perception of coordination across levels of care*** | | | | | | | | | | |
| I think that in this area patient care is coordinated between PC and SC doctors |  | 1,012(32.87) | 549(25.65) | **0.78(0.65-0.94**) | 339(31.59) | 215(24.18) | 0.81(0.61-1.08) | 673(33.55) | 334(26.70) | 0.78(0.68-0.91) |

^a^ Year of reference: 2017.

*^b^* the value corresponds to the categories *always/very often.*

*^c^* Adjusted by sex, level of care, years of experience as a doctor, type of hospital, type of specialty*.*

*^d^* Adjusted by sex, years of experience as a doctor, type of hospital, type of specialty

Variable type of area was used to adjust for a possible cluster effect.

*** Percentage of Don’t Know /No Answer higher than 30%

| \|  \|  \| **Total** \| \| \| **Primary care** \| \| \| **Secondary care** \| \| \| \| --- \| --- \| --- \| --- \| --- \| --- \| --- \| --- \| --- \| --- \| --- \| \|  \|  \| **2017**  **N=3308** \| **2022**  **N=2277** \| **2022/2017** ^a^ **changes** \| **2017**  **N=1141** \| **2022**  **N=945** \| **2022/2017** ^a^ **changes** \| **2017**  **N=2167** \| **2022**  **N=1332** \| **2022/2017** ^a^ **changes** \| \|  \|  \| **n (%)** \| **n (%)** \| a PR (CI 95%) ^f^ \| **n (%)** \| **n (%)** \| a PR (CI 95%) *^g^* \| **n (%)** \| **n (%)** \| a PR (CI 95%) *^g^* \|   **Supplementary table 2. Differences in access and frequency of use of coordination mechanisms between years, by level of care** | | | | | | | | | | |
| --- | --- | --- | --- | --- | --- | --- | --- | --- | --- | --- | --- | --- | --- | --- | --- | --- | --- | --- | --- | --- | --- | --- | --- | --- | --- | --- | --- | --- | --- | --- | --- | --- | --- | --- | --- | --- | --- | --- | --- | --- | --- | --- | --- |
| ***Shared EMR of Catalonia (HC3/HES)*** | | | | | | | | | | |
|  |  | N=3067 | N=1347 |  | N=1072 | N=509 |  | N=1995 | N=838 |  |
| Have access b |  | 3,057(99.67) | 1,335(99.48) | 1.00(0.99-1.00) | 1,068(99.63) | 504(99.41) | 0.99(0.99-1.00) | 1,989(99.70) | 831(99.52) | 1.00(0.99-1.00) |
| Frequent use c |  | 1,939(65.86) | 1,027(78.88) | **1.21(1.15-1.28)** | 802(77.26) | 442(90.20) | **1.17(1.14-1.19)** | 1,137(59.65) | 585(72.04) | **1.28(1.12-1.45)** |
| ***Shared EMR of the centre*** | | | | | | | | | | |
|  |  | N=2917 | N=1698 |  | N=1048 | N=671 |  | N=1869 | N=1027 |  |
| Have access b |  | 2,623(89.92) | 1,352(90.62) | 0.91(0.88-0.94) | 918(87.60) | 591(93.22) | 1.02(0.95-1.09) | 1,705(91.23) | 761(88.69) | 0.84(0.78-1.01) |
| Frequent use c |  | 2,044(81.27) | 1,171(88.51) | **1.07(1.03-1.11)** | 836(94.04) | 559(95.39) | 1.01(0.96-1.06) | 1,208(74.29) | 612(83.04) | **1.14(1.08-1.20)** |
| ***Electronic consultations through the EMR*** | | | | | | | | | | |
|  |  | N=2474 | N=1562 |  | N=1011 | N=537 |  | N=1463 | N=1025 |  |
| Have access b |  | 1,795(72.55) | 782(67.12) | **0.89(0.82-0.97)** | 841(83.18) | 397(81.86) | 0.93(0.88-0.98) | 954(65.21) | 385(56.62) | 0.91(0.80-1.06) |
| Frequent use d |  | 854(52.17) | 509(67.60) | **1.33 (1.25-1.41)** | 430(52.96) | 308(78.37) | **1.48(1.29-1.70)** | 424(51.39) | 201(55.83) | **1.13(1.08-1.18**) |
| ***Consultations through e-mail*** | | | | | | | | | | |
|  |  | N=2481 | N=1931 |  | N=996 | N=793 |  | N=1485 | N=1138 |  |
| Have access b |  | 1,885(75.98) | 836(56.79) | **0.72(0.69-0.79)** | 792(79.52) | 393(55.59) | **0.66(0.61-0.73)** | 1,093(73.60) | 443(57.91) | **0.77(0.64-0.92)** |
| Frequent used |  | 460(26.11) | 139(17.25) | **0.66(0.50-0.89)** | 179(23.31) | 59(15.40) | **0.59(0.43-0.83)** | 281(28.27) | 80(18.91) | 0.69(0.46-1.05) |
| ***Consultations through telephone*** | | | | | | | | | | |
|  |  | N=2615 | N=1798 |  | N=976 | N=716 |  | N=1639 | N=1082 |  |
| Have access b |  | 2,085(79.73) | 425(32.12) | **0.37(0.32-0.43)** | 700(71.72) | 160(25.60) | **0.36(0.30-0.44)** | 1,385(84.50) | 265(37.97) | **0.38(0.32-0.45)** |
| Frequent use d |  | 425(21.73) | 81(20.00) | 0.94(0.85-1.03) | 86(12.80) | 21(13.29) | 1.09(0.81-1.47) | 339(26.40) | 60(24.29) | 0.98(0.91-1.05) |
| ***Joint clinical case conferences*** | | | | | | | | | | |
|  |  | N=2817 | N=1891 |  | N=1045 | N=754 |  | N=1772 | N=1137 |  |
| Have access b |  | 1,226(43.52) | 298(17.89) | **0.37(0.32-0.43)** | 696(66.60) | 160(21.92) | **0.32(0.26-0.40)** | 530(29.91) | 138(14.74) | **0.45(0.44-0.47)** |
| Frequent use e |  | 748(62.49) | 167(57.79) | **0.89(0.79-0.98)** | 473(69.46) | 80(51.61) | 0.74(0.59-0.93) | 275(53.29) | 87(64.93) | 1.14(0.96-1.29) |

^a^ Year of reference: 2017.

^b^ the value corresponds to the category *Yes.*

The value corresponds to the category frequent use*: ^c^Daily, ^d^Daily or Weekly and, ^e^ Daily, Weekly or Monthly* within those who reported to have access.

^f^Adjusted by sex, years of experience as a doctor, level of care, type of specialty, type of hospital.

*^g^* Adjusted by sex, years of experience as a doctor, type of specialty, type of hospital.

Variable type of area was used to adjust for a possible cluster effect.

| **Supplementary table 3. Differences in factors related to clinical coordination between years, by level of care** | | | | | | | | | | |
| --- | --- | --- | --- | --- | --- | --- | --- | --- | --- | --- |
|  |  | **Total** | | | **Primary Care** | | | **Secondary care** | | |
|  |  | **2017**  **N=3308** | **2022**  **N=2277** | **2022/2017** ^a^ **changes** | **2017**  **N=3308** | **2022**  **N=2277** | **2022/2017** ^a^ **changes** | **2017**  **N=3308** | **2022**  **N=2277** | **2022/2017** ^a^ **changes** |
|  |  | **n (%)** *^b^* | **n (%)** *^b^* | a PR (CI 95%) *^c^* | **n (%)** *^b^* | **n (%)** *^b^* | a PR (CI 95%) *^d^* | **n (%)** *^b^* | **n (%)** *^b^* | a PR (CI 95%) *^d^* |
| ***Organizational factors*** | | | | | | | | | | |
| My organization’s management facilitates coordination between PC and SC doctors |  | 1,492(50.97) | 707(37.25) | **0.72(0.70-0.75)** | 665(65.52) | 412(52.28) | **0.80(0.77-0.82)** | 827(43.25) | 295(26.58) | **0.66(0.61.-0.72)** |
| My organization sets objectives that are aimed at coordination between care levels |  | 1,370(46.98) | 660(35.07) | **0.75(0.69-0.81)** | 540(53.20) | 317(40.59) | **0.77(0.69-0.86)** | 830(43.66) | 343(31.15) | **0.75(0.72-0.79)** |
| The time I can dedicate to coordinating with doctors of the other level during my working day is sufficient |  | 380(13.04) | 180(9.49) | **0.71(0.58-0.88)** | 136(13.47) | 82(10.42) | 0.76(0.55-1.05) | 244(12.82) | 98(8.84) | **0.69(0.60-0.79)** |
| ***Interactional factors between doctors*** | | | | | | | | | | |
| I know the doctors of the other care level who see my patients personally |  | 1,103(37.86) | 395(20.86) | **0.59(0.44-0.73)** | 442(43.68) | 180(22.90) | **0.53(0.48-0.58)** | 661(34.77) | 215(19.40) | **0.61(0.44-0.84)** |
| I trust in the clinical skills of the doctors of the other level who see my patients |  | 2,429(83.30) | 1,547(81.77) | 0.95(0.91-1.00) | 971(96.04) | 733(93.26) | 0.96(0.92-1.02) | 1,458(76.54) | 814(73.60) | **0.94(0.90-0.99)** |
| My daily practice influences the practice of the doctors of the other level |  | 1,834(62.94) | 1,176(62.03) | 1.02(0.96-1.10) | 554(54.85) | 450(57.25) | 1.08(0.92-1.28) | 1,280(67.23) | 726(65.41) | 1.00(0.96-1.04) |
| In practice, primary care doctors are responsible for coordinating the patients on their way through the different care levels |  | 2,189(75.22) | 1,432(75.45) | 0.99(0.96-1.02) | 950(93.87) | 706(89.59) | **0.96(0.94-0.98)** | 1,239(65.28) | 726(65.41) | 1.03(0.98-1.09) |
| **Attitudinal factors** | | | | | | | | | | |
| Satisfaction with the job in the organization ^e^ |  | 2.193(75.70) | 1.262(66.91) | **0.88(0.86-0.90)** | 741(74.17) | 533(68.25) | **0.91(0.87-0.96)** | 1.452(76.50) | 729(65.97) | **0.86(0.81-0.92)** |

^a^ Year of reference: 2017.

*^b^* the value corresponds to the categories *always/very often*

*^c^* Adjusted by sex, level of care, years of experience as a doctor, type of hospital, type of specialty*.*

*^d^* Adjusted by sex, years of experience as a doctor, type of hospital, type of specialty. Variable type of area was used to adjust for a possible cluster effect.

^e^ the value corresponds to the category: yes
